# Emotion Dysregulation and Mentalization in Adult Attention-Deficit/Hyperactivity Disorder

**DOI:** 10.3390/brainsci16070679

**Published:** 2026-06-27

**Authors:** Alberto Gabbiadini, Gabriele Avincola, Clarissa Fichera, Giuliana Maccarone, Ludovico Mineo, Alessandro Rodolico, Emi Bondi, Maria Salvina Signorelli

**Affiliations:** 1 Psychiatry Unit, Department of Clinical and Experimental Medicine, University of Catania, 95123 Catania, Italy; 2 Department of Psychiatry, ASST Papa Giovanni XXIII, 24100 Bergamo, Italy

**Keywords:** adult attention-deficit/hyperactivity disorder (ADHD), emotion dysregulation, reflective functioning, mentalization, executive dysfunction, mentalization-based therapy (MBT)

## Abstract

**Highlights:**

**What are the main findings?**

- Adults with ADHD showed impaired reflective functioning and emotion dysregulation.
- Reflective functioning uncertainty is consistent with a possible indirect pathway ADHD symptom severity and emotion dysregulation.

**What are the implications of the main findings?**

- Mentalizing deficits may contribute to emotion dysregulation in ADHD.
- Reflective functioning may represent a therapeutic target in ADHD.

**Abstract:**

**Purpose:** This study examined the relationships among attention-deficit/hyperactivity disorder (ADHD) symptoms, emotion dysregulation, and reflective functioning (RF) in adults. Specifically, it explored whether reflective functioning uncertainty—defined as difficulties in understanding one’s own and others’ mental states—was associated with the relationship between ADHD symptom severity and emotion dysregulation. **Methods:** In this case–control observational study, 40 adults with ADHD and 40 healthy controls completed the Adult ADHD Self-Report Scale (ASRS), the Difficulties in Emotion Regulation Scale (DERS), and the Reflective Functioning Questionnaire (RFQ). Analyses included group comparisons, correlation analyses, linear regression models, and adjusted mediation analysis. **Results:** Compared with controls, adults with ADHD showed significantly greater emotion dysregulation and higher reflective functioning uncertainty. ADHD symptom severity was positively associated with both emotion dysregulation and reflective functioning uncertainty. Mediation analysis supported a significant indirect effect, whereas the direct effect was not statistically significant, a pattern consistent with a possible indirect pathway. **Conclusions:** These findings suggest that difficulties in reflective functioning may be associated with the relationship between ADHD symptoms and emotion dysregulation in adults with ADHD. Assessing emotion dysregulation and reflective functioning may improve the clinical characterization of adult ADHD. Further studies with larger samples, longitudinal designs, and multimethod assessment are needed to clarify the directionality and clinical implications of these relationships.

## 1. Introduction

Attention-deficit/hyperactivity disorder (ADHD) is a neurodevelopmental condition typically emerging in childhood and characterized by inattention, hyperactivity, and impulsivity [1]. Approximately half of individuals diagnosed in childhood continue to experience clinically significant symptoms into adulthood, although their expression often differs from that observed in younger populations [2]. Adults with ADHD frequently report inner restlessness, impulsivity, impatience, excessive talkativeness, and difficulties with planning and organization. Functional impairments may include occupational instability, interpersonal difficulties, disorganization, procrastination, distractibility, and difficulty sustaining motivation [3].

The variability of symptom presentation in adulthood often complicates differential diagnosis, particularly with mood disorders, bipolar disorder, and borderline personality disorder. Diagnostic assessment is further complicated by the high prevalence of psychiatric comorbidities, including mood, anxiety, personality, and substance use disorders [4].

In recent years, emotion dysregulation has received increasing attention as a clinically relevant dimension of ADHD, potentially complementing the traditional symptom domains of inattention, hyperactivity, and impulsivity [5]. Defined as difficulties in monitoring, evaluating, and modulating emotional responses to achieve adaptive functioning [6], emotion dysregulation is associated with substantial psychosocial impairment and is increasingly recognized as an important component of ADHD symptomatology [7].

Research in personality disorders has linked emotion dysregulation to mentalization, operationalized in research as Reflective Functioning (RF), the capacity to interpret one’s own and others’ behavior in terms of underlying mental states such as thoughts, emotions, desires, and beliefs, and to integrate these representations into coherent self- and interpersonal understanding [8,9,10].

Reflective functioning describes the ability to interpret behavior in terms of underlying mental states such as thoughts, emotions, desires, and beliefs, and to integrate these representations into coherent self- and interpersonal understanding [9].

Empirical research on mentalization in ADHD remains limited. Some studies suggest that individuals with ADHD may exhibit reduced mentalizing capacity, potentially associated with executive dysfunction and attentional impairments. For example, Akça [11] reported that both ADHD and borderline personality disorder are associated with mentalization difficulties, although with different patterns, while earlier work has linked ADHD to reduced theory of mind performance. In adults, Perroud et al. [10] reported impaired reflective functioning in ADHD and an association between mentalizing difficulties and emotion dysregulation, particularly difficulties in anger regulation.

Recent systematic reviews have substantially advanced the understanding of emotional and social cognitive difficulties in adult ADHD. Soler-Gutiérrez et al. [12] conducted a comprehensive review documenting that adults with ADHD consistently demonstrate greater use of maladaptive emotion regulation strategies compared to controls, and that emotion dysregulation is associated with symptom severity, executive dysfunction, and psychiatric comorbidities. Complementarily, Morellini et al. [13] reviewed evidence of social cognition impairments in adult ADHD across domains including emotion recognition, Theory of Mind, empathy, and decision-making, highlighting the relevance of social-cognitive processes for interpersonal functioning in this population. However, neither of these reviews addressed RF as a specific construct, nor examined whether mentalizing capacity might serve as a mechanism linking ADHD symptom severity to emotion dysregulation. The relationship between RF uncertainty and emotion dysregulation, and its potential role as a mediating factor in adult ADHD, thus remains unexplored. The present study aims to address this gap by directly examining reflective functioning in relation to emotion dysregulation within a case–control design and by exploring whether reflective functioning uncertainty may be associated with a possible indirect pathway linking ADHD symptom severity and emotion dysregulation.

Based on this background, the present study had three main aims: (1) to examine differences in emotion dysregulation and reflective functioning between adults with ADHD and healthy controls; (2) to investigate the associations among ADHD symptom severity, emotion dysregulation, and reflective functioning in both groups; and (3) to explore whether reflective functioning uncertainty was associated with the relationship between ADHD symptom severity and emotion dysregulation through an exploratory mediation model.

Based on previous findings, we hypothesized that adults with ADHD would exhibit greater emotion dysregulation and higher reflective functioning uncertainty than healthy controls. We further expected ADHD symptom severity to be positively associated with emotion dysregulation and reflective functioning uncertainty, and negatively associated with reflective functioning certainty. Finally, we hypothesized that reflective functioning uncertainty would be associated with the relationship between ADHD symptom severity and emotion dysregulation, and we explored whether this pattern was consistent with a possible indirect pathway.

## 2. Methods

### 2.1. Study Design

This was a case–control observational study comparing adults with ADHD and healthy controls. Data were collected from January 2022 at the outpatient services of the Psychiatric Unit of Policlinico G. Rodolico in Catania, Italy, and the Adult ADHD Outpatient Clinic of ASST Papa Giovanni XXIII in Bergamo, Italy. Control participants were recruited from the general population.

### 2.2. Participants

A total of 40 adults with ADHD and 40 healthy controls were included in the study. Inclusion criteria for the ADHD group were age ≥ 18 years and a clinical diagnosis of ADHD confirmed by a structured diagnostic interview according to DSM-5 criteria. Psychiatric comorbidities were allowed and mainly included mood and anxiety disorders. Control participants were aged ≥ 18 years and had no current or past psychiatric diagnosis based on self-reported psychiatric history obtained during recruitment. No structured psychiatric interview was administered to the control group.

Individuals with severe neurological disorders, intellectual disability, or other conditions that could impair the ability to understand the study procedures, complete the questionnaires, or provide informed consent were excluded. All participants provided written informed consent prior to participation. Sociodemographic information, psychiatric history, and medication status were collected. The study was approved by the Ethics Committee of the University of Catania (2021/No. 3) and conducted in accordance with the Declaration of Helsinki.

### 2.3. Instruments

The patients underwent clinical and psychodiagnostic evaluation (DIVA-5) that confirmed the diagnosis of ADHD in adults according to DSM-5 criteria. Both groups underwent psychometric evaluation with the following instruments: ASRS, DERS, and RFQ.

#### 2.3.1. The Diagnostic Interview for ADHD in Adults (DIVA-5)

The DIVA is based on DSM-V criteria and is the first Dutch structured interview for ADHD in adults. The DIVA was developed by J.J.S. Kooij and M.H. Francken and is the follow-up version to the Semi-structured Interview for ADHD in adults. In order to make it easier to verify the presence or absence of the 18 symptomatic criteria of ADHD, both in childhood/adolescence and adulthood, the interview provides a list of concrete and realistic examples referring to both current and past (childhood/adulthood) behavior. These examples are taken from the descriptions given most frequently by adult patients in clinical practice. Some concrete examples are also taken from the difficulties typically associated with symptoms in five areas of daily life: work and education, romantic and family relationships, social relationships, leisure and hobbies, self-esteem, and self-image [14].

#### 2.3.2. Adult Attention-Deficit/Hyperactivity Disorder Self-Reporting Rating Scale (ASRS)

The ASRS scale is a self-administered questionnaire consisting of 18 questions, validated for subjects older than 18 years concerning the specific symptoms of ADHD. It is divided in two parts: Part A contains 6 items, which has been found to be the most predictive of ADHD and are best for use as a screening instrument. Part B contains 12 additional questions based on DSM criteria that provide additional cues and can serve as further probes into the patient’s symptoms [15]. For a patients’ symptoms to be considered consistent with an ADHD diagnosis, they require 4 or more responses at specific severity levels in Part A of the ASRS. Items 5, 6, 12, 13, 14, 15, 16, 17, and 18 identify symptoms from the hyperactivity cluster; the remaining items refer to the inattention sphere.

#### 2.3.3. The Reflective Functioning Questionnaire, Summary Version (RFQ)

The Italian version [16] of the RFQ, originally developed by Fonagy et al. in 2016 [8], was employed to assess reflective functioning. This tool aims to investigate the self-reported tendency to consider information about intentional mental states relevant for understanding one’s own and others’ behaviors. It consists of eight items, scored on a seven-point Likert scale. The scoring procedure provides two subscales measuring Certainty and Uncertainty about mental state information, that is, how certain or uncertain individuals are about whether actions are intrinsically intentional or motivated by internal mental states such as emotions, thoughts, or needs. Regarding the items of the Certainty scale (e.g., “I don’t always know why I do what I do”), low agreement reflects hyper-mentalizing, while high agreement reflects more genuine mentalizing (acknowledging the opacity of mental states).

As for the Uncertainty scale (e.g., “Sometimes I do things without really knowing why”), agreement with the items distinguishes between individuals who have an almost complete lack of understanding of mental states and those who recognize the complexity and opacity of their own and others’ mental states, indicative of genuine mentalizing [8,17].

#### 2.3.4. The Difficulties in the Emotion Regulation Scale (DERS)

The DERS is a short 36-item self-administered questionnaire designed to assess multiple aspects of emotion dysregulation. The measure provides a total score and six scales derived from factor analysis: 1. non-acceptance of emotional responses; 2. difficulty in engaging in goal-directed behavior; 3. difficulty in impulse control; 4. lack of emotional awareness; 5. limited access to emotion regulation strategies; 6. lack of emotional clarity. DERS was examined in a sample of college students aged 18 and older, and in a clinical sample of women with borderline personality disorder [18].

### 2.4. Statistical Analysis

All analyses were conducted using SPSS version 29 and StataNow 19. Statistical significance was set at *p* < 0.05 (two-tailed). Descriptive statistics included means, standard deviations, medians, interquartile ranges, and frequencies. Distribution normality was assessed using the Shapiro–Wilk test. Between-group differences in continuous variables were evaluated using the Mann–Whitney U test, and categorical variables using Pearson’s chi-square test. Effect sizes for continuous variables were calculated as Rosenthal’s r derived from standardized rank statistics, and for categorical variables, as Cramer’s V.

Spearman’s rank correlation coefficients (ρ) were used as exploratory measures of bivariate association and were therefore not adjusted for demographic or clinical covariates. Potential confounding effects were subsequently addressed within the adjusted mediation analyses. Correlation strength was interpreted according to conventional guidelines: small (0.10–0.29), medium (0.30–0.49), and large (≥0.50) [19].

Linear regression analyses were conducted within each group to examine associations among ADHD symptom severity (ASRS), reflective functioning uncertainty, and emotion dysregulation (DERS). Covariates were selected a priori based on their potential influence on emotion dysregulation and reflective functioning and their frequent use as adjustment variables in adult ADHD research. Age and sex were included as demographic factors potentially associated with both emotion dysregulation and mentalizing abilities. To facilitate interpretation of the adjusted regression analyses, corresponding unadjusted models (excluding age and sex as covariates) were additionally estimated and compared with the adjusted models. Standardized regression coefficients (β) and associated *p* values were reported.

An exploratory mediation analysis was conducted within the ADHD group to examine whether reflective functioning uncertainty was associated with the relationship between ADHD symptom severity (ASRS) and emotion dysregulation (DERS). The significance of the indirect effect was tested using the Sobel test [20]. Since preliminary findings suggested that reflective functioning Uncertainty was consistent with a possible indirect pathway, the association between ADHD symptoms and emotion dysregulation, given the known limitations of normal-theory approaches for estimating indirect effects, particularly in relatively small samples, the mediation model was subsequently confirmed using a bootstrap procedure.

A mediation analysis based on structural equation modeling with 10,000 bootstrap resamples was conducted. ADHD symptom severity (ASRS total score) was entered as the independent variable, emotion dysregulation (DERS total score) as the dependent variable, and reflective functioning uncertainty subscale score as the mediator. To account for potential confounding factors, age, sex, pharmacological treatment, and psychiatric comorbidities were included as covariates in the model.

To evaluate the adequacy of the sample size for detecting mediation effects, a post-hoc Monte Carlo power analysis for indirect effects was performed using the observed correlation structure and the Monte Carlo Power Analysis for Indirect Effects application [21]. Given the cross-sectional design, mediation results were interpreted cautiously and not considered evidence of causal relationships [22].

To address alternative theoretical explanations, an additional mediation model was explored in which emotion dysregulation was specified as the mediator between ADHD symptom severity and reflective functioning uncertainty.

The potential influence of concurrent psychotropic medication use and psychiatric comorbidities was explored through additional subgroup nonparametric analyses (Mann–Whitney U test). Psychotropic medications included anxiolytics, mood stabilizers, antidepressants, antipsychotics, and methylphenidate; due to the small number of participants receiving medications, it was coded as a binary variable (any medication vs. none). Psychiatric comorbidity was similarly coded as a dichotomous variable indicating the presence or absence of at least one additional psychiatric diagnosis. Participants were stratified according to medication status and the presence of psychiatric comorbidity. Group differences in ASRS total, DERS total, and RFQ Uncertainty scores were evaluated using the Mann–Whitney U test and reported as median and interquartile range.

## 3. Results

### 3.1. Sociodemographic and Clinical Characteristics of the Sample

As shown in Table 1, a total of 80 participants were included in the study: 40 patients with ADHD and 40 healthy controls. The ADHD sample consisted of 65.0% males and 35.0% females, whereas the control group included 52.5% males and 47.5% females. The difference in sex distribution between groups was not statistically significant (χ^2^ = 1.29, *p* = 0.256; Cramer’s V = 0.13), indicating only a small between-group effect and suggesting substantial comparability in sex distribution.

Regarding continuous variables, normality assumptions were assessed using the Shapiro-Wilk test. Within the ADHD group, all variables showed normal distributions (*p* ≥ 0.05), except for the ASRS Inattentive and RFQ Certainty subscales and age. In the control group, all examined variables showed non-normal distributions. Therefore, non-parametric tests were used for group comparisons.

The mean age of patients with ADHD was 28.25 years (range: 18–52; median = 24.50), whereas controls had a mean age of 27.38 years (range: 19–42; median = 27.00). The Mann–Whitney U test showed no statistically significant difference between groups (U = 735.00, *p* = 0.530; r = 0.07), indicating a negligible effect size and supporting demographic comparability between groups.

For the remaining continuous variables (ASRS, DERS, and RFQ), Mann–Whitney U tests showed statistically significant differences between ADHD patients and healthy controls (all *p* < 0.001), with consistently large effect sizes (Table 1). Specifically, the ADHD group showed higher ASRS total scores (median = 13.00 vs. 3.00), as well as higher Hyperactivity (median = 5.00 vs. 1.00) and Inattention scores (median = 8.00 vs. 1.50). Regarding emotion dysregulation, patients with ADHD reported significantly higher DERS scores (median = 118.00) compared with controls (median = 65.50). reflective functioning, assessed through the RFQ Certainty and Uncertainty subscales, also differed significantly between groups (all *p* < 0.001), with higher RFQ Uncertainty and lower RFQ Certainty scores observed among participants with ADHD.

Within the ADHD group, 16 participants (40.0%) were receiving psychotropic medication, and 15 (37.5%) presented at least one psychiatric comorbidity. Additional subgroup analyses were conducted within the ADHD sample to examine whether psychotropic treatment status or psychiatric comorbidity were associated with the main study variables (Supplementary Table S1). Group comparisons using Mann–Whitney U tests did not reveal statistically significant differences in ASRS total, DERS total, or RFQ Uncertainty scores according to medication status or the presence of psychiatric comorbidity (all *p* > 0.05). These findings suggest that neither psychotropic treatment nor overall psychiatric burden substantially influenced the main study variables within the present sample. Therefore, both variables were subsequently included as covariates in the adjusted mediation analysis to account for potential clinical confounding.

### 3.2. Correlations Between RFQ Uncertainty, DERS, ASRS, Age, and Gender

Correlations between variables are reported in Table 2. In the ADHD group, correlations are shown in the bottom left cells, under the diagonal of the table.

A large effect size (r ≥ 0.50) can be observed between RFQ Uncertainty and the variables RFQ Certainty, DERS, and ASRS total scores. Medium effect sizes are observed between RFQ Uncertainty and both ASRS Hyperactivity (0.482, *p* = 0.002) and ASRS Inattention (0.370, *p* = 0.019).

Spearman’s rank correlation coefficient for the RFQ Certainty subscale showed an opposite trend compared to the Uncertainty subscale: correlations with the other clinical variables were all negative. The only significant correlations were with DERS (−0.474, *p* = 0.002) and the ASRS Inattention subscale (−0.334, *p* = 0.035). A medium effect size can be observed between DERS and ASRS total score (0.464, *p* = 0.003), ASRS Inattention (0.404, *p* = 0.010), and Hyperactivity (0.411, *p* = 0.008) subscales. The ASRS total score shows a large effect size with its two subscales, ASRS Hyperactivity and Inattention (0.913 and 0.655, respectively, *p* < 0.001). These two subscales relate to each other with a medium effect size (0.325, *p* = 0.040).

In the control group, a negative large effect size can be observed between the two RFQ subscales (−0.598, *p* < 0.001). RFQ Uncertainty also shows a medium effect size with DERS (0.383, *p* = 0.015), ASRS (0.352, *p* = 0.026), and the Inattentive subdimension of ASRS (0.336, *p* = 0.034). RFQ Certainty appears to correlate negatively with DERS, showing a large effect size (−0.618, *p* < 0.001). Additionally, there is a negative medium effect size between RFQ Certainty and ASRS (−0.416, *p* = 0.008), as well as with its Inattentive subdimension (−0.418, *p* = 0.007). DERS displays a positive medium effect size with ASRS (0.402, *p* = 0.010) and its Inattentive subdimension (0.451, *p* = 0.004). The most significant results in the control group are those between ASRS total and its subscales scores: Hyperactivity (0.630, *p* < 0.001) and Inattention (0.941, *p* < 0.001). These two subscales correlate with a medium effect size (0.389, *p* = 0.013).

In conclusion, the most notable findings from the Spearman’s rank correlations in the ADHD group are those between RFQ Uncertainty and DERS and between RFQ Uncertainty and ASRS. In the control group, the most significant correlation is between RFQ Certainty and DERS. We did not consider it valuable to thoroughly examine the correlations between a total score variable (e.g., ASRS total score) and its subcomponents (e.g., ASRS Hyperactivity subscale), or between subcomponents of the same scale.

### 3.3. Linear Regression Between ASRS Total, DERS and RFQ Uncertainty

Linear regression analyses were performed to further investigate the relationships among RFQ Uncertainty, ASRS total score, and DERS scores, which showed the strongest associations in the Spearman correlation analyses within the ADHD group. Table 3 summarizes the regression models examining the associations between ASRS total and RFQ Uncertainty, ASRS total and DERS, and RFQ Uncertainty and DERS, respectively, as independent and dependent variables.

To account for potential demographic confounding, age and sex were included as covariates in the regression models. Sex showed a significant association only in models predicting RFQ Uncertainty in both groups, whereas age did not significantly contribute to the regression models. Given the limited and non-uniform contribution across outcomes, these findings should be interpreted cautiously. To facilitate interpretation of the adjusted analyses, we additionally compared these models with the corresponding unadjusted regressions. Excluding age and sex did not meaningfully alter the magnitude, direction, or statistical significance of the observed associations in either the ADHD or control groups. In particular, the association between RFQ Uncertainty and DERS remained stable in the ADHD group (unadjusted B = 23.65, *p* < 0.001) and in the control group (unadjusted B = 32.41, *p* = 0.001), indicating that the association between RFQ Uncertainty and DERS scores was not primarily explained by demographic differences between groups. Additional adjustment for psychiatric comorbidity and pharmacological treatment was subsequently implemented within the mediation analysis.

Among participants with ADHD, all regression models yielded significant associations between the variables of interest. Specifically, higher ADHD symptom severity was associated with greater uncertainty in reflective functioning and higher levels of emotion dysregulation, whereas greater reflective functioning uncertainty was strongly associated with emotion dysregulation. In contrast, no significant associations emerged in the control group, apart from the relationship between RFQ Uncertainty and DERS, which remained significant independently of ADHD symptoms. This finding suggests that deficits in reflective functioning may be associated with emotion dysregulation beyond ADHD diagnosis alone.

### 3.4. Correlation of DERS and ASRS Total with RFQ Uncertainty as a Mediator

To further investigate the relationship between ADHD symptom severity, reflective functioning, and emotion dysregulation, an exploratory mediation analysis was initially performed using the Sobel test. Preliminary findings suggested that reflective functioning was consistent with a possible indirect pathway between ADHD symptoms and emotion dysregulation. However, given the known limitations of normal-theory approaches for estimating indirect effects, particularly in relatively small samples, the mediation model was subsequently confirmed using a bootstrap procedure.

**Figure 1:**
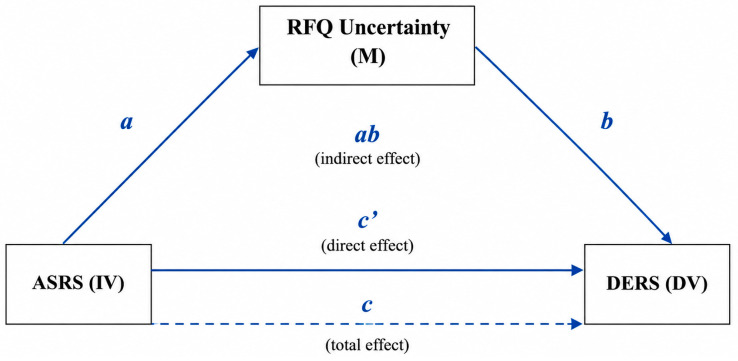
Mediation model with ADHD severity as the independent variable (IV), emotion dysregulation as the dependent variable (DV), and deficit of reflective functioning as the mediator (M). The mediation model examines the indirect effect of reflective functioning uncertainty on the association between ADHD severity (ASRS total score) and emotion dysregulation (DERS total score). Path a indicates the association between ASRS and RFQ Uncertainty; path b indicates the association between RFQ Uncertainty and DERS; path c′ represents the direct effect of ASRS on DERS; path ab represents the indirect effect through RFQ Uncertainty; and path c represents the total effect of ASRS on DERS.

A mediation analysis based on structural equation modeling with 10,000 bootstrap resamples was conducted. ADHD symptom severity (ASRS total score) was entered as the independent variable, emotion dysregulation (DERS total score) as the dependent variable, and reflective functioning uncertainty as the mediator (Figure 1). To account for potential confounding factors, age, sex, pharmacological treatment, and psychiatric comorbidities were included as covariates in the model.

Results showed that ADHD symptoms significantly predicted reflective functioning uncertainty (B = 0.12, *p* < 0.001), while the latter was significantly associated with emotion dysregulation (B = 26.25, *p* < 0.001). The total effect of ADHD symptoms on emotion dysregulation was significant (B = 3.43, *p* = 0.001); however, after the inclusion of reflective functioning in the model, the direct effect was markedly reduced and no longer statistically significant (B = 0.19, *p* = 0.828). Importantly, the indirect effect remained significant after adjustment for covariates (B = 3.24, BootSE = 0.87, 95% CI [1.54, 4.94]), with the confidence interval not including zero. The main mediation results are presented in Table 4; the complete adjusted model, including demographic and clinical covariates, is provided in Supplementary Table S2.

Overall, these findings support the hypothesis that impairments in reflective functioning is compatible with a possible indirect pathway between ADHD symptom severity and emotion dysregulation independently of demographic and clinical covariates. This pattern is consistent with a model in which emotion dysregulation, rather than representing an independent dimension of ADHD, may be at least partially explained by deficits in the capacity to reflect upon and regulate internal emotional states.

Given the relatively small sample size, an additional post-hoc Monte Carlo power analysis for indirect effects was performed to evaluate whether the mediation model was adequately powered. Using the observed correlations among ADHD symptom severity, reflective functioning uncertainty, and emotion dysregulation in the ADHD sample (N = 40), the estimated statistical power to detect the indirect effect was 0.93. These findings suggest that the available sample size was adequate to detect the observed mediation effect, although replication in larger independent samples remains warranted.

To address alternative theoretical explanations, an additional mediation model was explored in which emotion dysregulation was specified as the mediator between ADHD symptom severity and reflective functioning uncertainty. Although a significant indirect effect emerged, the direct association between ADHD symptoms and reflective functioning uncertainty remained statistically significant, suggesting that this alternative model may reflect both direct and indirect associations (Supplementary Table S3).

## 4. Discussion

This study examined reflective functioning and emotion dysregulation in adults with ADHD compared with healthy controls. Participants with ADHD showed significantly higher RFQ Uncertainty subscale and DERS scores than controls, suggesting greater difficulties in both mentalizing processes and emotion regulation. A high RFQ Uncertainty score reflects reduced confidence in understanding mental states underlying one’s own and others’ behavior [10]. However, because the RFQ is a self-report measure, it cannot distinguish between impaired recognition of mental states and reduced reliance on mental state information, as described in some personality disorders [10]. Higher DERS scores indicate greater emotion regulation difficulties. Overall, these findings are consistent with previous literature reporting emotion dysregulation and a tendency toward hypo-mentalization in ADHD. In addition, participants with ADHD showed lower RFQ Certainty scores, suggesting reduced engagement in mental state thinking when interpreting behavior.

Within the ADHD group, symptom severity was positively associated with both reflective functioning uncertainty and emotion dysregulation. This pattern is consistent with a link between ADHD symptom burden, reduced mentalizing, and greater emotion regulation difficulties. One possible interpretation is that executive dysfunction, commonly observed in ADHD, may contribute to reduced capacity to process and integrate mental state information [23].

Difficulties in mentalization may be particularly relevant in ADHD because attentional deficits, impulsivity, and executive dysfunction can interfere with the ability to monitor and interpret one’s own and others’ mental states. Reduced reflective functioning may in turn hinder the recognition and modulation of emotional experiences, increasing vulnerability to emotional reactivity and difficulties in emotion regulation. This conceptual framework provides a plausible explanation for the observed associations among ADHD symptom severity, reflective functioning uncertainty, and emotion dysregulation.

Although our study did not investigate biological mechanisms directly, it may be useful to consider broader neurocognitive frameworks that could underlie both executive dysfunction and mentalizing impairments. A broader neurobiological perspective may help contextualize these findings. Although ADHD is primarily considered a neurodevelopmental disorder, executive dysfunction and cognitive regulation deficits are influenced by molecular mechanisms involved in neuronal development, synaptic plasticity, and neural network functioning.

Among these mechanisms, alternative splicing, a post-transcriptional process essential for generating neuronal protein diversity and maintaining synaptic function, has emerged as a key regulator of cognitive performance and neuronal integrity. Dysregulation of alternative splicing has been increasingly implicated in several neurodegenerative disorders, including Alzheimer’s disease, Parkinson’s disease, amyotrophic lateral sclerosis, and frontotemporal dementia, where it contributes to cognitive decline and executive dysfunction through alterations in genes involved in synaptic transmission, neuronal maintenance, and network stability [24].

Although the present study did not investigate molecular mechanisms and ADHD differs substantially from neurodegenerative conditions in terms of etiology and developmental course, this comparative perspective highlights how disruptions in fundamental biological pathways may contribute to vulnerabilities in higher-order cognitive processes across different brain disorders. Such a framework may be useful for future research exploring shared neurobiological mechanisms underlying executive and mentalizing deficits in ADHD and other neuropsychiatric conditions.

Advancing this perspective, future research may benefit from adopting systemic and multimodal neurobiological approaches that integrate molecular, neural, and behavioral levels of analysis. Emerging evidence suggests that combining brain imaging with molecular markers, such as proteomic profiles, can help identify brain–body pathways linking emotional and cognitive functioning to broader physiological and metabolic processes. For example, the integration of neuroimaging and plasma proteomic data has recently revealed neurobiological signatures connecting depression and metabolic dysfunction, involving alterations in frontal and limbic brain networks as well as systemic metabolic pathways [25]. Although this work focused on depression, its methodological approach may provide a useful framework for ADHD research. Multimodal strategies combining neuroimaging with molecular markers, including proteomic and metabolomic measures, could help clarify the biological mechanisms underlying executive dysfunction, mentalizing difficulties, and emotional dysregulation in ADHD. More broadly, these approaches recognize that psychiatric symptoms are unlikely to result from isolated neural abnormalities alone, but rather from complex interactions between brain networks, peripheral biological systems, and broader regulatory processes. While such methodologies remain largely unexplored in ADHD, they may contribute to a more comprehensive understanding of neuropsychiatric vulnerability and help identify shared brain–body mechanisms across different psychiatric conditions.

Concerning reflective functioning, in the present sample RFQ Uncertainty scores were positively associated with emotion dysregulation in both groups, although the association appeared stronger in the ADHD group, suggesting a close relationship between mentalizing difficulties and emotion regulation independent of diagnostic status. Conversely, RFQ Certainty was negatively associated with DERS scores in both groups, indicating that greater reflective functioning was related to lower emotion dysregulation. Interestingly, RFQ Certainty was not associated with overall ADHD severity but showed a negative association with inattention symptoms, a finding that warrants further investigation.

Mediation analysis indicated that reflective functioning uncertainty was significantly associated with the relationship between ADHD symptom severity and emotion dysregulation. The indirect effect was significant, whereas the direct effect was not statistically significant, a pattern consistent with a possible indirect pathway. These findings suggest that mentalizing difficulties may be associated with emotion dysregulation in adults with ADHD. Specifically, greater uncertainty in understanding one’s own and others’ mental states was associated with higher levels of emotion dysregulation and may represent one possible pathway through which ADHD symptom severity relates to emotional difficulties. One possible interpretation is that reduced capacity to interpret and integrate internal emotional experiences may be associated with less effective emotion regulation strategies in adults with ADHD.

However, because this study used a cross-sectional design, the direction of the observed relationships cannot be established, and these findings should not be interpreted as evidence of a causal mechanism. Alternative models remain plausible: emotion dysregulation could mediate the association between ADHD symptoms and reduced reflective functioning, rather than the reverse, or a common factor such as executive dysfunction could influence both simultaneously. To address this issue, we additionally explored an alternative mediation model in which emotion dysregulation was specified as the mediator (Supplementary Table S3). Although a significant indirect effect emerged, the direct association between ADHD symptom severity and reflective functioning uncertainty remained statistically significant, suggesting the coexistence of direct and indirect associations rather than a fully indirect pattern.

We specified the primary model based on previous theoretical and longitudinal evidence suggesting that mentalizing capacity may influence emotion regulation processes over time, supporting reflective functioning as a theoretically plausible antecedent. This interpretation is further consistent with findings showing that ineffective mentalizing is associated with emotion dysregulation across both clinical and non-clinical samples [26]. However, longitudinal studies in ADHD populations are needed to test these competing models directly.

From a clinical perspective, these findings may help clarify the overlap between ADHD and other conditions characterized by emotion dysregulation, such as borderline personality disorder. Assessing reflective functioning may provide additional information beyond core ADHD symptoms by identifying difficulties in understanding and interpreting mental states that contribute to interpersonal problems, emotional instability, and impaired self-regulation. From a practical standpoint, routine clinical assessment of adults with ADHD could benefit from including brief measures of reflective functioning, such as the Reflective Functioning Questionnaire (RFQ), alongside standard ADHD and emotion dysregulation measures.

Identifying individuals with elevated RFQ Uncertainty scores may help clinicians recognize patients who are at greater risk of emotion dysregulation and interpersonal difficulties and who may require a more comprehensive therapeutic approach. In terms of intervention planning, mentalization-based approaches, originally developed for borderline personality disorder [27] may represent a promising adjunctive strategy for adults with ADHD presenting with significant mentalizing difficulties. Such interventions could be integrated with established treatments, including psychoeducation, executive function training, and pharmacotherapy, with the aim of improving emotional awareness, interpersonal functioning, and emotion regulation [28].

Although longitudinal and experimental studies are needed to confirm their clinical utility in ADHD, future treatment research could examine whether improvements in reflective functioning are associated with corresponding reductions in emotion dysregulation, thereby providing more direct evidence for the role of reflective functioning as a clinically relevant treatment target.

### 4.1. Limitations

This study has several limitations. First, the relatively modest sample size may have limited statistical power, reduced the ability to detect smaller effects or demographic interactions, and constrained the generalizability of the findings. Although no statistically significant differences in age and sex distribution emerged between groups and effect sizes for these variables were small, subtle contributions of demographic factors to the observed associations cannot be excluded. Therefore, replication in larger and more diverse samples is warranted to further evaluate the role of age and sex in these relationships.

Second, all major constructs, ADHD symptom severity, emotion dysregulation, and reflective functioning were assessed exclusively through self-report questionnaires rather than clinician-administered or performance-based measures. Although the RFQ and DERS represent widely used and validated measures, reliance on self-report questionnaires may introduce common bias and increase the influence of subjective reporting tendencies. This issue may be particularly relevant in ADHD populations, where executive dysfunction, reduced self-monitoring abilities, and difficulties in metacognitive appraisal could affect the accuracy of self-perceived emotional and mentalizing capacities.

This consideration is especially pertinent for the interpretation of the RFQ Uncertainty subscale, which reflects perceived difficulties in understanding one’s own and others’ mental states rather than directly measuring reflective functioning performance. Therefore, higher RFQ Uncertainty scores may partly capture subjective uncertainty or reduced insight rather than objective deficits in mentalization. Future studies should integrate self-report measures with performance-based and clinician-rated assessments of reflective functioning to validate these findings. Potential approaches include ecologically oriented mentalization paradigms such as the Movie for the Assessment of Social Cognition (MASC) [29], interview-based assessments of reflective functioning such as the Reflective Functioning Scale derived from the Adult Attachment Interview [30], and complementary experimental measures of social cognitive inference such as the Reading the Mind in the Eyes Test (RMET) [31].

Although mediation was initially explored using the Sobel test, the findings were subsequently confirmed using bootstrap estimation to improve robustness. Nevertheless, the cross-sectional nature of the study does not permit causal inference, and the observed mediation pattern should be interpreted as consistent with a possible indirect pathway rather than a definitive mechanistic process.

In addition, psychiatric comorbidities were collected as a dichotomous variable at the time of study design. Similarly, psychotropic treatment was modeled as a dichotomous variable for statistical analyses, although descriptive information regarding medication classes was additionally reported. Consequently, the present study could not examine the potential differential contribution of specific psychiatric diagnoses, medication classes, polypharmacy, dosage, or treatment duration to the observed associations.

Finally, the study focused primarily on the RFQ Uncertainty dimension to index hypo-mentalization, and future research should examine both RFQ dimensions and broader measures of mentalizing to better characterize reflective functioning in ADHD.

### 4.2. Future Directions

Future research should address the limitations of cross-sectional designs by adopting longitudinal and multimethod approaches capable of tracking changes in ADHD symptoms, emotion dysregulation, and reflective functioning over time. Such designs would allow for a more rigorous examination of temporal relationships, developmental trajectories, and the potential directionality of associations among these constructs. Ecological momentary assessment (EMA) may be particularly valuable in this regard, as it enables the real-time assessment of emotional states, mentalizing processes, and symptom fluctuations in naturalistic settings, thereby capturing dynamic within-person processes that retrospective self-report measures may fail to detect [32]. For example, EMA studies could investigate whether momentary increases in ADHD symptoms are associated with concurrent reductions in reflective functioning and whether such changes subsequently contribute to episodes of emotion dysregulation. These approaches may help clarify the temporal mechanisms linking executive dysfunction, mentalizing difficulties, and emotion dysregulation, providing a stronger empirical basis for the development of mechanism-informed interventions.

In addition, future EMA protocols could incorporate the systematic assessment of acute environmental stressors—such as interpersonal conflicts, academic or occupational demands, work-related pressures, and other unexpected daily challenges—alongside fluctuations in ADHD symptoms and reflective functioning. Because these stressors frequently act as proximal triggers of emotional dysregulation in adults with ADHD, examining their occurrence in real time may help elucidate how contextual factors interact with symptom severity and mentalizing capacity, thereby shaping the emergence and intensity of emotional dysregulation in everyday life.

These approaches could be further strengthened through integration with digital phenotyping methods and wearable technologies (e.g., smartphones and smartwatches), which allow the continuous collection of ecological behavioral and physiological data. Parameters such as activity levels, sleep patterns, heart rate variability, geolocation-derived routines, and real-time behavioral engagement may provide complementary information on contextual factors associated with fluctuations in ADHD symptoms and emotion regulation processes. Combined with EMA, these tools may contribute to a more ecologically valid and temporally sensitive characterization of dynamic interactions among executive functioning, emotional experiences, and mentalizing-related processes.

Integrating EMA with clinician-administered assessments, neurobiological measures, informant reports, or performance-based measures of mentalizing could help overcome some of the limitations inherent in exclusive reliance on self-report questionnaires, including reporting bias and shared method variance. Such multimethod approaches would provide a more comprehensive and ecologically valid understanding of the relationships among ADHD symptoms, emotion dysregulation, and reflective functioning in adults with ADHD.

## 5. Conclusions

In conclusion, the present findings highlight the relevance of assessing emotion dysregulation and reflective functioning in adults with ADHD. Incorporating these domains into the clinical evaluation may help refine the clinical characterization of emotional and interpersonal difficulties in adult ADHD. The observed associations between symptom severity, reflective functioning uncertainty, and emotion dysregulation suggest that mentalizing difficulties may be associated with the emotion profile of adults with ADHD, although causal relationships cannot be inferred.

Interventions targeting reflective functioning, such as mentalization-based approaches, may represent a promising area for future investigation; however, their clinical utility in ADHD requires confirmation in longitudinal and treatment studies. Further research with larger samples and multimethod assessment approaches, including ecologically valid and repeated measures of emotional and mentalizing processes, is needed to better understand the relationships among ADHD symptoms, emotion dysregulation, and reflective functioning.

## Figures and Tables

**Table 1:** Sociodemographic and clinical characteristics of the sample.

Effect Size (r)	*p* Value	Test	Control (*n* = 40)	ADHD (*n* = 40)	Continuous Variables
0.070	0.530	U = 735.00	27.00 [24.00–29.00]	24.50 [22.00–34.50]	Years of age
0.811	<0.001	U = 48.50	3.00 [1.25–4.00]	13.00 [10.00–15.00]	ASRS total score
0.710	<0.001	U = 147.50	1.00 [0.00–1.75]	5.00 [3.00–7.00]	ASRS Hyperactivity subscale score
0.796	<0.001	U = 65.50	1.50 [1.00–3.00]	8.00 [6.25–8.00]	ASRS Inattention subscale score
0.756	<0.001	U = 97.50	65.50 [55.25–84.75]	118.00 [99.25–129.50]	DERS total score
0.726	<0.001	U = 138.00	0.00 [0.00–0.33]	1.17 [0.83–1.63]	RFQ Uncertainty subscale score
0.662	<0.001	U = 187.50	2.00 [1.25–2.50]	0.25 [0.00–0.83]	RFQ Certainty subscale score
-	-	-	-	16 (40.0%)	Any psychotropic medication, *n* (%)
-	-	-	-	15 (37.5%)	Psychiatric comorbidity, *n* (%)
**Effect size (V)**	***p* value**	**Test**	**Control** **(*n* = 40)**	**ADHD** **(*n* = 40)**	**Categorical variable**
0.127	0.256	χ^2^(1) = 1.29	21 (52.5)	26 (65.0)	Gender (male), *n* (%)

Note. Values are presented as median [interquartile range] unless otherwise indicated. Continuous variables were compared using the Mann–Whitney U test and effect sizes are reported as r derived from standardized rank statistics. Categorical variables were compared using Pearson’s chi-square test and effect sizes are reported as Cramer’s V. U = Mann–Whitney U statistic; ASRS = Adult ADHD Self-Report Scale; DERS = Difficulties in Emotion Regulation Scale; RFQ = Reflective Functioning Questionnaire. Psychotropic medications included anxiolytics, mood stabilizers, antidepressants, antipsychotics, and methylphenidate.

**Table 2:** Spearman correlations among clinical variables by group.

ASRS Inattention	ASRS Hyperactivity	ASRS Total	DERS Total	RFQ Certainty	RFQ Uncertainty	Variable
0.38 *	0.06	0.35 *	0.38 *	−0.60 ***	—	RFQ Uncertainty
−0.42 **	−0.24	−0.42 **	−0.62 ***	—	−0.76 ***	RFQ Certainty
0.45 **	0.13	0.40 *	—	−0.47 **	0.72 ***	DERS total
0.94 ***	0.63 ***	—	0.46 **	−0.26	0.52 ***	ASRS total
0.39 *	—	0.91 ***	0.41 **	−0.15	0.48 **	ASRS Hyperactivity
—	0.33 *	0.66 ***	0.40 **	−0.33 *	0.37 *	ASRS Inattention

Note. Spearman’s rho coefficients are shown. Lower triangle reports correlations for the ADHD group (*n* = 40); upper triangle reports correlations for the control group (*n* = 40). *p* < 0.05 *, *p* < 0.01 **, *p* < 0.001 ***. RFQ = Reflective Functioning Questionnaire; DERS = Difficulties in Emotion Regulation Scale; ASRS = Adult ADHD Self-Report Scale.

**Table 3:** Exploratory linear regression models within groups.

ADHD Group (*n* = 40)				
*p*	β	B (SE)	Predictor	Dependent Variable
0.009	0.52	0.11 (0.03)	ASRS total	RFQ Uncertainty
0.044	0.30	0.42 (0.20)	Gender	
0.068	−0.24	−0.02 (0.01)	Age	
0.36			Model R^2^	
0.001	0.53	3.53 (0.94)	ASRS total	DERS total
0.843	0.03	1.35 (6.77)	Gender	
0.181	−0.21	−0.54 (0.39)	Age	
0.26			Model R^2^	
0.000	0.80	25.30 (3.55)	RFQ Uncertainty	DERS total
0.094	−0.20	−9.24 (5.38)	Gender	
0.825	0.03	0.07 (0.30)	Age	
0.59			Model R^2^	
**Control group (*n* = 40)**				
0.115	0.26	0.02 (0.02)	ASRS total	RFQ Uncertainty
0.044	0.20	0.14 (0.09)	Gender	
0.653	0.24	0.00 (0.01)	Age	
0.14			Model R^2^	
0.102	0.28	1.66 (0.68)	ASRS total	DERS total
0.902	0.10	0.72 (1.10)	Gender	
0.895	−0.12	0.09 (0.18)	Age	
0.07			Model R^2^	
0.001	0.52	34.33 (9.86)	RFQ Uncertainty	DERS total
0.485	−0.11	−3.81 (5.40)	Gender	
0.844	0.03	0.12 (0.58)	Age	
0.25			Model R^2^	

Note. Unstandardized regression coefficients (B) are reported with standard errors in parentheses. β indicates standardized coefficients. All models include age and gender as covariates. Analyses are exploratory and were conducted separately within each group. RFQ = Reflective Functioning Questionnaire; DERS = Difficulties in Emotion Regulation Scale; ASRS = Adult ADHD Self-Report Scale.

**Table 4:** Mediation analysis examining the role of reflective functioning in the relationship between ADHD symptoms and emotion dysregulation.

95% CI	*p*	z	SE (Boot)	B	Effect	Path
0.07, 0.18	<0.001	4.30	0.03	0.12	ASRS → RFQ Uncertainty	a
18.81, 33.70	<0.001	6.91	3.80	26.25	RFQ Uncertainty → DERS	b
−1.52, 1.90	0.828	0.22	0.87	0.19	ASRS → DERS (direct effect)	c′
1.54, 4.94	<0.001	3.73	0.87	3.24	ASRS → RFQ Uncertainty → DERS (indirect effect)	ab
1.38, 5.48	0.001	3.28	1.05	3.43	ASRS → DERS (total effect)	c

Note. B = unstandardized regression coefficient; SE (Boot) = bootstrap standard error; CI = confidence interval. Mediation analysis was conducted using structural equation modeling with 10,000 bootstrap resamples. Age, sex, pharmacological treatment, and psychiatric comorbidities were included as covariates.

## Data Availability

The raw data supporting the conclusions of this article will be made available by the authors on request.

## Supplementary Materials

The following supporting information can be downloaded at: https://www.mdpi.com/article/10.3390/brainsci16070679/s1, Table S1: Clinical characteristics of the ADHD sample; Table S2: Mediation model adjusted for demographic and clinical covariates; Table S3: Alternative mediation model with emotion dysregulation as mediator.
