# Supplementary material for: Emotion Dysregulation and Mentalization in Adult Attention-Deficit/Hyperactivity Disorder
Source: Brain Sci. 2026 Jun 27;16(7):679. doi: 10.3390/brainsci16070679 (PMC13406483; doi:10.3390/brainsci16070679)
Supplement: Supplementary file 1 [file brainsci-16-00679-s001.zip › brainsci-4341891-supplementary.pdf]

MDPI

brain sciences

Supplementary materials

Supplementary Table S1. - Clinical characteristics of the ADHD sample

Panel A. Clinical characteristics	
**Variable**	**ADHD sample (N = 40)**
Psychiatric comorbidity present, n (%)	15 (37.5)
Psychotropic treatment present, n (%)	16 (40.0)
**Medication class**	
Mood stabilizers	8 (20.0)
Antidepressants	5 (12.5)
Antipsychotics	2 (5.0)
Anxiolytics	3 (7.5)
Methylphenidate	5 (12.5)
Combination treatment*	4 (10.0)

Panel B. Exploratory comparison according to psychotropic treatment				
**Outcome**	**No treatment (n = 24)**	**Treatment (n = 16)**	**z**	**p**
ASRS total	14 [9.50-15.00]	10.5 [10.00-14.00]	1.03	.303
DERS total	119.5 [103.50-133.50]	105.5 [98.00-125.50]	0.84	.400
RFQ Uncertainty	1.08 [0.67-1.50]	1.17 [0.83-1.58]	-0.85	.396

Panel C. Exploratory comparison according to psychiatric comorbidity				
**Outcome**	**No comorbidity (n = 25)**	**Comorbidity (n = 15)**	**z**	**p**
ASRS total	14 [10.00-15.00]	10 [10.00-14.00]	1.25	.210
DERS total	121 [104.00-132.00]	103 [97.00-124.00]	1.47	.142
RFQ Uncertainty	1.17 [0.83-1.67]	1.17 [0.83-1.50]	-0.44	.662

Note. Values are expressed as n (%) in Panel A and as median [interquartile range] in Panels B and C unless otherwise specified. Psychiatric comorbidity was coded as a dichotomous variable (present/absent). Medication classes were coded as non-mutually exclusive categories; therefore, category totals may exceed the number of treated participants. Continuous variables were compared using the Mann–Whitney U test.

*Combination treatment refers to the concurrent use of more than one psychotropic medication.

Supplementary Table S2. - Mediation model adjusted for demographic and clinical covariates

**Outcome**	**Predictor**	**B**	**SE (Boot)**	**z**	**p**	**95% CI**
RFQ Uncertainty	ASRS total	0.12	0.03	4.30	<.001	0.07, 0.18
	Age	-0.02	0.01	-2.22	.027	-0.05, <0.00
	Sex	0.32	0.21	1.51	.131	-0.10, 0.74
	Pharmacological treatment	0.37	0.38	0.97	.331	-0.38, 1.12
	Psychiatric comorbidity	0.08	0.36	0.23	.819	-0.63, 0.80
DERS total	RFQ Uncertainty	26.25	3.80	6.91	<.001	18.81, 33.70
	ASRS total (direct effect)	0.19	0.87	0.22	.828	-1.52, 1.90
	ASRS total (indirect effect)	3.24	0.87	3.73	<.001	1.54, 4.94
	ASRS total (total effect)	3.43	1.05	3.28	.001	1.38, 5.48
	Age	0.17	0.60	0.65	.515	-0.34, 0.68
	Sex	-7.16	4.80	-1.49	.136	-16.57, 2.24
	Pharmacological treatment	-5.26	7.65	-0.69	.491	-20.25, 9.72

 https://doi.org/10.3390/xxxxx

Outcome	Predictor	B	SE (Boot)	z	p	95% CI
	Psychiatric comorbidity	-9.04	8.35	-1.08	.279	-25.41, 7.32

Note. B = unstandardized regression coefficient; SE (Boot) = bootstrap standard error; CI = confidence interval. Mediation analysis was conducted using structural equation modeling with 10,000 bootstrap resamples. Reflective functioning uncertainty (RFQ Uncertainty) was specified as the mediator between ADHD symptom severity (ASRS total) and emotional dysregulation (DERS total). Age, sex, pharmacological treatment, and psychiatric comorbidities were included as binary covariates. Coefficients are reported as unstandardized estimates.

Supplementary Table S3. – Alternative mediation model with emotion dysregulation as mediator

Effect	B	SE (Boot)	z	p	95% CI
Total effect (ASRS → RFQ Uncertainty)	0.12	0.03	4.26	<.001	0.07, 0.18
Direct effect (ASRS → RFQ Uncertainty)	0.05	0.03	2.02	.043	0.00, 0.10
Indirect effect (ASRS → DERS → RFQ Uncertainty)	0.07	0.03	2.83	.005	0.02, 0.12

Note. B = unstandardized regression coefficient; SE (Boot) = bootstrap standard error; CI = confidence interval. Mediation analysis was conducted using structural equation modeling with 10,000 bootstrap resamples. Emotion dysregulation (DERS total) was specified as the mediator between ADHD symptom severity (ASRS total) and reflective functioning uncertainty (RFQ Uncertainty). Age, sex, pharmacological treatment, and psychiatric comorbidities were included as binary covariates. Coefficients are reported as unstandardized estimates.

https://doi.org/10.3390/xxxxx
